# A superfolder variant of pH-sensitive pHluorin for *in vivo* pH measurements in the endoplasmic reticulum

**DOI:** 10.1038/s41598-018-30367-z

**Published:** 2018-08-10

**Authors:** Mara Reifenrath, Eckhard Boles

**Affiliations:** Institute of Molecular Biosciences, Faculty of Biological Sciences, Goethe University Frankfurt, Max-von-Laue Straße 9, 60438 Frankfurt am Main, Germany

## Abstract

Many cellular processes are regulated via pH, and maintaining the pH of different organelles is crucial for cell survival. A pH-sensitive GFP variant, the so-called pHluorin, has proven to be a valuable tool to study the pH of the cytosol, mitochondria and other organelles *in vivo*. We found that the fluorescence intensity of Endoplasmic Reticulum (ER)-targeted pHluorin in the yeast *Saccharomyces cerevisiae* was very low and barely showed pH sensitivity, probably due to misfolding in the oxidative environment of the ER. We therefore developed a superfolder variant of pHluorin which enabled us to monitor pH changes in the ER and the cytosol of *S*. *cerevisiae in vivo*. The superfolder pHluorin variant is likely to be functional in cells of different organisms as well as in additional compartments that originate from the secretory pathway like the Golgi apparatus and pre-vacuolar compartments, and therefore has a broad range of possible future applications.

## Introduction

A tight regulation of the pH in the cytosol as well as in different organelles is crucial for many cellular processes. This also holds true for the secretory pathway where the luminal pH acidifies continuously: the pH of the Endoplasmic Reticulum (ER) is known to be near neutral, the Golgi apparatus is slightly acidic and secretory granules have been shown to reach pH values of about 5.2^1^.

pH can strongly impact protein conformation and enzyme activity by influencing ionization states of acidic and basic amino acid side chains. In the secretory pathway the stepwise acidification is crucial for sorting of proteins and posttranslational modifications^1,2^. Various studies show the pH dependency of ER to Golgi transport^3–5^ and Golgi to ER retrieval of ER-resident proteins^6–9^. Also the sugar recognition of cargo receptors in the early secretory pathway is pH dependent^10^. Results of Kellokumpu *et al*.^11^ suggest that alterations of Golgi pH may cause abnormal glycosylation which is a common phenotypic change in human malignant cells. Furthermore, studies on the papillomavirus E5 oncoprotein^12^ and on the influenza virus M_2_ protein^13–16^ show that both induce alkalinization of the Golgi as an important step in cellular transformation and virus assembly, respectively. The above mentioned studies demonstrate the importance of pH maintenance in the different compartments of the secretory pathway, and reveal that pH alterations can contribute to the pathology of certain diseases.

A large number of tools to measure intracellular pH have been developed including the use of pH-sensitive fluorescent dyes^17^, ^31^P NMR^18,19^ and benzoic acid as a pH tracer^20^. A very limited number of tools allows accessing the pH of specific organelles including 2′,7′-*bis*(carboxyethyl)-5(6)-carboxyfluorescein which accumulates in yeast vacuoles^21^, pH-sensitive fluorophores covalently bound to shiga-like toxins targeting Golgi or ER^22,23^ and pH-sensitive fluorescein-biotin in combination with avidin-chimera proteins targeted to ER or Golgi^24^.

So far, the genetically encoded pH-sensitive GFP variant pHluorin^25^ is one of the most convenient and easiest ways to measure intracellular pH. It has been used to study cytosolic pH^26–30^ as well as organellar pH, including mitochondria^31^ and peroxisomes^32^. pHluorin was used in various organisms including yeast^31,33–35^. Here, we show that the original pHluorin is not suitable for measuring the pH of *S. cerevisiae* ER, probably due to misfolding in the oxidative environment of this organelle. To overcome this problem, we developed a superfolder variant of pHluorin which allows reliable and straight-forward pH determination in the ER.

## Results and Discussion

### Superfolder pHluorin shows improved pH sensitivity and fluorescence intensity in the ER of *S*. *cerevisiae* cells

**Figure 1:**
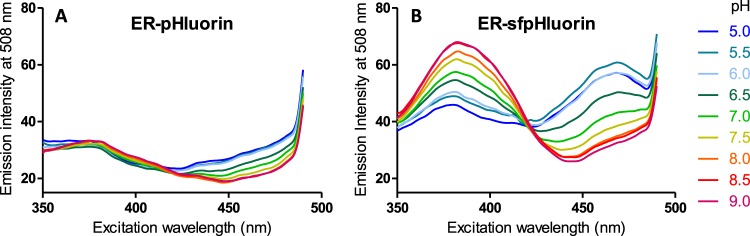
pH dependence of excitation spectra of ER-pHluorin and superfolder ER-pHluorin (ER-sfpHluorin). Excitation spectra of ER-pHluorin (**A**) and superfolder ER-pHluorin (**B**) expressing *S*. *cerevisiae* cells. The cells were permeabilized with digitonin and resuspended in citric acid/Na_2_HPO_4_ buffer of pH values ranging from 5.0 to 9.0 to an OD_600_ of 0.5. The emission intensity was recorded at 508 nm.

Ratiometric pHluorin targeted to the ER of *S*. *cerevisiae* (ER-pHluorin) by fusing the ER signal sequence of Kar2 to its N-terminus and an ER retention tag containing the amino acid sequence HDEL to its C-terminus showed very low fluorescence intensity (Fig. 1A) compared to cytosolically located pHluorin (Supplementary Fig. S1). Furthermore, the signal was almost independent of the pH (Fig. 1A) and did not allow for pH analysis in the ER. A superfolder GFP variant^36^ was reported to be fluorescent in oxidizing environments^37^ in contrast to enhanced GFP. Therefore, we aimed to construct a superfolder pHluorin by combining pHluorin mutations^25^ with the ones of the superfolder GFP^36^. As pHluorin was constructed by several rounds of mutagenesis, it is not known which of the pHluorin mutations are actually necessary for its pH sensitivity^25^. We transferred the majority of the pHluorin mutations to the superfolder GFP sequence, excluding the mutations E132D and L220F (for full sequence of sfpHluorin see Supplementary Table S1). The E132D mutation was excluded as this amino acid is not closely located to any histidine residue and thereby is not likely be involved in pH sensing. The pHluorin mutation L220F was excluded as a pHluorin construct without this mutation was previously shown to have maintained its pH sensitivity^31^. Superfolder pHluorin targeted to the ER by fusing it to the ER signal sequence of Kar2 and the HDEL ER retention tag, showed significantly increased fluorescence intensities and a clear pH sensitivity (Fig. 1B) in contrast to the original pHluorin targeted to the ER (Fig. 1A).

**Figure 2:**
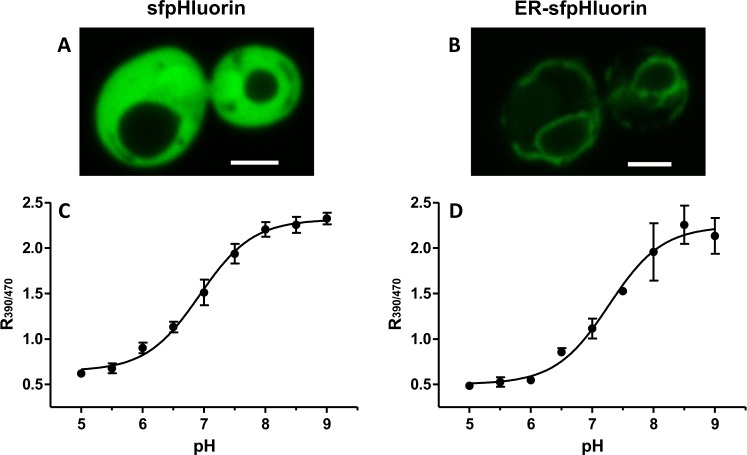
Intracellular localization and pH calibration of sfpHluorin and ER-sfpHluorin. Fluorescence microscopy images of sfpHluorin (**A**) and ER-sfpHluorin (**B**) in *S. cerevisiae* cells. Scale bars correspond to 2 µm. pH calibration with permeabilized *S*. *cerevisiae* cells expressing sfpHluorin (**C**) and ER-sfpHluorin (**D**). The emission intensity at 512 nm with an excitation wavelength of 390 nm was divided through the emission intensity (512 nm) with an excitation wavelength of 470 nm yielding the ratio R_390/470_. Mean and standard deviation of biological triplicates are shown (mean ± s.d.). The graphical nonlinear fit (sigmoidal dose-response) was performed with the GraphPad Prism software. The best-fit values and the standard error of the fit can be found in the Supplementary Table S4A and B.

Next, we analyzed the intracellular localization of the superfolder ER-pHluorin construct to exclude that the increased fluorescence signal might originate from a mislocalization to a different compartment. We compared the ER-targeted construct (ER-sfpHluorin) to superfolder pHluorin without targeting signals (sfpHluorin). sfpHluorin showed a clear cytosolic localization (Fig. 2A) whereas ER-sfpHluorin was found in the typical ER pattern (Fig. 2B)^38–41^. Nevertheless, it cannot be completely excluded that a very minor fraction of ER-sfpHluorin is still located in other compartments like the cytosol or Golgi.

Having successfully constructed a superfolder pHluorin which can be functionally expressed both in the cytosol and the ER we prepared a calibration curve using a microplate reader (Fig. 2C,D) to allow for the simultaneous analysis of 96 samples. We calibrated from pH 5–9 with cells expressing sfpHluorin (Fig. 2C) and ER-sfpHluorin (Fig. 2D).

### Superfolder pHluorin enables measuring the pH of cytosol and ER during growth and starvation

**Figure 3:**
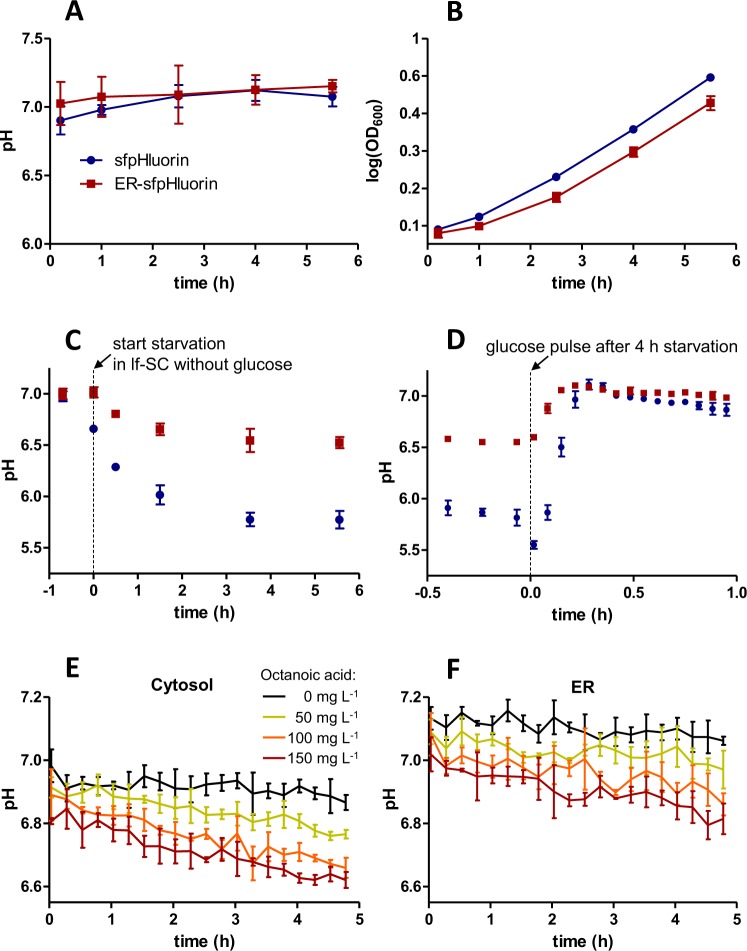
pH of cytosol and ER of *S*. *cerevisiae* during growth, starvation, following a glucose pulse and upon octanoic acid addition. (**A** and **B**) *S*. *cerevisiae* cells expressing sfpHluorin (blue) or ER-sfpHluorin (red) grown in lf-SCD medium with 0.1 mM methionine (slightly repressible conditions) were reinoculated in fresh lf-SCD with 0.1 mM methionine to an OD_600_ of 0.1. The pH (**A**) of cytosol and ER and the OD_600_ (**B**) were analyzed during 6 hours after reinoculation. (**C** and **D**) *S*. *cerevisiae* cells expressing sfpHluorin (blue) or ER-sfpHluorin (red) were grown into the exponential growth phase in lf-SCD medium without methionine (fully induced conditions), washed and resuspended in lf-SC medium without methionine and without glucose. pH during glucose starvation (**C**) and after a glucose pulse (2% w/v) after 4 hours of starvation (**D**) was followed. (**E** and **F**) The pH of cytosol (**E**) and ER (**F**) was followed after addition of 0 mg L^−1^ (black), 50 mg L^−1^ (yellow), 100 mg L^−1^ (orange) and 150 mg L^−1^ (red) octanoic acid. Mean and standard deviation of biological triplicates are shown (mean ± s.d.).

The new superfolder pHluorin variants allowed us to follow the pH of both cytosol and ER during cell growth (Fig. 3A,B) and upon glucose starvation (Fig. 3C). When growing in the presence of glucose *S*. *cerevisiae* cells maintained a pH of 7.1 in the ER, very similar to the cytosolic pH under the same conditions (Fig. 3A). Surprisingly, we recorded that after resuspension of the cells in lf-SC medium without glucose, the pH of the cytosol dropped faster and to a lower value than the pH of the ER (Fig. 3C). 3.5 hours after glucose starvation the cytosolic pH stabilized at 5.8 whereas the pH in the ER stabilized at 6.5. Both did not change within the following 2 hours. Several studies in which the pH of the ER was investigated concluded that the ER is highly proton permeable and that it has no active proton pumps^23,24,42^. However, our results indicate that at least during glucose starvation, the ER membrane has either a decreased proton permeability, or that somehow resident proton pumps or proton pumps during ER transit get activated.

We also analyzed how the pH of the cytosol and the ER of glucose starved cells change after a glucose pulse (Fig. 3D). Various studies showed the phenomenon of fast initial acidification and subsequent alkalinization of the cytosol of glucose starved *S*. *cerevisiae* after a glucose pulse^20,31,43^. Here we show with the use of ER-sfpHluorin, that the pH of the ER behaves similarly as the pH of the cytosol after a glucose pulse. Within few minutes both the pH of the cytosol and the ER increase up to a near neutral pH. An initial acidification after the glucose pulse was only detected for the cytosol, however we cannot exclude that the effect was present in the ER as well, but was not discovered due to the small timescale.

### Octanoic acid leads to an acidification of *S*. *cerevisiae* cytosol and ER

Octanoic acid is a byproduct of *S*. *cerevisiae* and is known to inhibit fermentation and growth at concentrations even lower than 1 mM^44–46^. Octanoic acid is also important for biotechnological purposes and yeast cells producing increased amounts of octanoic acid are currently being developed in our laboratory^47^; however, the toxicity of octanoic acid imposes some problems. Viegas and Sá-Correia^44^ showed that addition of octanoic acid decreases cytosolic pH of *S*. *cerevisiae* in the exponential growth phase. We tested the effect of octanoic acid to both, the cytosol and the ER. Starting with cells from the same precultures growing in the exponential growth phase we added different amounts of octanoic acid (0, 50, 100, 150 mg L^−1^) at time 0 and followed the pH for 5 hours (Fig. 3E,F). The medium was buffered (40 mM KH_2_PO_4_, pH 6.3) so that the initial pH was independent of the octanoic acid concentration. Anyhow, its acidifying effect to the cells was detectable immediately after octanoic acid addition. Already at the first recorded timepoint (2 min after octanoic acid addition) the pH of the cytosol dropped approximately 0.2 pH units from 7.0 to 6.8 upon addition of 150 mg L^−1^ octanoic acid. The effect on the ER was similar ‒ the pH immediately dropped about 0.1 pH units from 7.1 to 7.0. These results suggest, that either octanoic acid enters the cells and causes a drop in pH or that it causes membrane leakage and an approximation of the intracellular pH to the pH of the medium. The latter theory is supported by results of Liu *et al*.^45^ who showed that octanoic acid induces membrane leakage in *S*. *cerevisiae* and Borrull *et al*.^46^ who showed that octanoic acid mainly accumulates in the plasma membrane fraction. The high proton permeability of the ER membrane^23,24,42^ is most likely to have caused the pH drop in the ER.

### Conclusion and Outlook

In this study, we developed and tested a pHluorin variant which is strongly improved for measuring pH of the ER of *S*. *cerevisiae*. Most likely, the original pHluorin misfolds in the ER due to the oxidative environment which we successfully circumvented by combining mutations of superfolder GFP and original pHluorin. With superfolder pHluorin, we could reliably follow the pH of the ER and the cytosol of *S*. *cerevisiae* during growth, glucose starvation, after a glucose pulse and upon addition of octanoic acid.

The superfolder pHluorin can be used in the future to study pH changes in the ER under certain growth conditions or in mutant strains. For strains carrying mutations expected to influence ER sorting or to induce ER stress the localization of ER-sfpHluorin should be analyzed again to exclude that observed pH changes originate from mislocalization of the probe.

Superfolder pHluorin is likely to also show improved properties in certain compartments other than the ER. pHluorin targeted to downstream compartments of the secretory pathway like the Golgi, COPII- or COPI-coated vesicles and pre-vacuolar compartments, would need to pass through the ER as well. Therefore, it is very likely that the superfolder pHluorin we present here will also show improved performance compared to original pHluorin when used in additional compartments of the secretory pathway, and not only in yeast but also in other eukaryotic cells. Moreover, it might also enable measuring pH changes in the oxidative environment of the bacterial periplasm.

## Methods

### Strains

*S*. *cerevisiae* strain CEN.PK2-1C^48^ was used for cloning, pH calibration and intracellular pH measurements. The strain was always freshly transformed on solid SCD medium (as described in Bruder *et al*.^49^, with 2% glucose) omitting uracil. For subcloning of plasmids *E*. *coli* strain DH10B (Gibco BRL, Gaithersburg, MD) was used.

### Plasmid construction

All plasmids used in this study are listed in Supplementary Table S2.

For assembly of the plasmids the vector backbone p426MET25^50^ was linearized using restriction enzymes *Bam*HI and *Sal*I (New England Biolabs GmbH). For plasmid construction by homologous recombination (according to Gietz and Schiestl^51^) the linearized vector backbone was used together with the corresponding PCR fragments to transform CEN.PK2-1C.

PCRs were performed with Phusion polymerase (New England Biolabs GmbH) and primers as listed in Supplementary Table S3. The gene of pHluorin was amplified from the vector pYES-PACT1-pHluorin (as published in Orij *et al*.^31^ a kind gift from Dr. Gertien Smits).

We compared the sequence of superfolder GFP (Pédelacq *et al*.^36^, a kind gift from Dr. Frank Bernhard) with the sequence of the pHluorin construct. The sequences of pHluorin and superfolder GFP, as used in this study, are stated in Supplementary Table S1. To create the superfolder pHluorin variant, we introduced the following mutations into the superfolder GFP variant: T65S, S147E, N149L, I161T, N164I, K166Q, I167V, R168H and S202H. This was done by amplifying superfolder GFP gene with primers JTP96 and MRP85, MRP86 and MRP87, MRP88 and MRP89, MRP90 and JTP97 (Supplementary Table S3) to yield 5 PCR products containing corresponding overhangs to each other. The mutations E132D and L220F of the pHluorin gene were not included in the superfolder pHluorin variant. The sequence of the superfolder pHluorin variant we constructed is stated in Supplementary Table S1.

For proteins targeted to the ER, the sequence encoding the ER signal sequence of Kar2 (amino acids 1–42) was amplified from genomic DNA of CEN.PK2-1C and introduced upstream of the (superfolder) pHluorin coding sequence. Additionally, the sequence encoding a C-terminal ER retention signal containing the motif HDEL was added (coding sequences in Supplementary Table S1 and primers in Supplementary Table S3).

### Strain cultivation

CEN.PK2-1C transformants were cultivated in low fluorescence synthetic complete medium (lf-SCD) containing 6.9 g L^−1^ YNB with ammonium sulfate, without amino acids, without folic acid and without riboflavin (MP Biomedicals), 2% w/v glucose and amino acids as stated in Bruder *et al*.^49^, uracil was omitted. The medium was filter-sterilized. The octanoic acid test was performed in buffered low fluorescence synthetic minimal medium (lf-SMD) containing 6.9 g L^−1^ YNB with ammonium sulfate, without amino acids, without folic acid and without riboflavin (MP Biomedicals) and 2% w/v glucose, 0.093 mM tryptophan, 0.439 mM leucine and 0.124 mM histidine for auxotrophic requirements. The medium was buffered with 40 mM KH_2_PO_4_ (pH 6.3). All pHluorin variants were under control of the methionine repressible promoter of *MET25* (*MET25*_*prom*_). If not stated otherwise the medium contained 0 mM methionine resulting in a strong expression of the pHluorin variants.

### Fluorescence excitation spectra and pH calibration of pHluorin

Fluorescence excitation spectra were recorded with a fluorescence spectrometer (LS55, Perkin Elmer) to visualize the pH dependence and differences between ER-pHluorin and ER-sfpHluorin. To generate a calibration for analysis of a larger set of samples, we used a Clariostar plate reader (BMG LABTECH GmbH).

In both cases the cells were grown in 5 ml lf-SCD and were harvested in the exponential growth phase (at an OD_600_ between 1.0 and 2.0). The treatment with 100 µg digitonin ml^−1^ to permeabilize the cells was done according to Orij *et al*.^31^. After washing, cells were resuspended in PBS (137 mM NaCl, 2.7 mM KCl, 10 mM Na_2_HPO_4_, 1.8 mM KH_2_PO_4_, pH 7.4) to an OD_600_ of 20 and put on ice. The cells were then added to citric acid/Na_2_HPO_4_ buffer of pH values ranging from 5.0 to 9.0 to an OD_600_ of 0.5.

The excitation spectra for the different pH values were recorded in 2 ml quarz cuvettes at room temperature using the LS55 fluorescence spectrometer (settings: λ_em_ = 508 nm, slit 15 nm; λ_es_ = 350 to 490 nm, slit 10 nm; scan speed 500 nm min^−1^). The experiment was done in biological triplicates, out of which the spectra of one sample are shown.

To generate the calibration curves, we used a Clariostar microplate reader (BMG LABTECH GmbH) set to 30 °C and black polystyrene clear-bottom 96-well microtitre plates (Greiner Bio One, article Nr. 655097). Fluorescence emission was measured at 512 nm (±10 nm) with excitation bands of 9 nm centered around of 390 nm and 470 nm. The same settings were used for pH analysis during cell growth, after a glucose pulse and after addition of octanoic acid. The calibration was done with biological triplicates. We performed the calibration twice for sfpHluorin and ER-sfpHluorin – once with cells grown in medium containing 0 mM methionine (fully inducible conditions) (Fig. 2C,D) and once with cells grown in medium containing 0.1 mM methionine (slightly repressible conditions) (Supplementary Fig. S2A,B). We used the former one to analyze the pH in cells grown in medium without methionine (Fig. 3C–F: starvation, glucose pulse and octanoic acid pulse) and the latter one to follow the pH of both cytosol and ER during exponential growth in lf-SCD with 0.1 mM methionine (Fig. 3A,B). The raw data used for the calibrations was blank corrected, the blank being CEN.PK2-1C cells harboring an empty vector (p426MET25).

### Intracellular pH measurements

To follow the pH of the cytosol and the ER during growth precultures in biological triplicates were grown in lf-SCD containing 0.1 mM methionine until the late exponential phase (OD_600_ between 2.0 and 3.0). The cultures were then reinoculated in pre-warmed lf-SCD containing 0.1 mM methionine in Erlenmeyer flasks. The cells were grown at 30 °C and 180 rpm, and samples were taken at 30 °C and immediately transferred to the plate reader which was set to 30 °C.

For the starvation and glucose pulse experiment, biological triplicates were grown overnight in lf-SCD (0 mM methionine) and harvested in the exponential phase (OD_600_ between 1.0 and 2.0). To analyze the initial pH before starvation, cells were resuspended in their own growth medium to an OD_600_ of 0.9 and analyzed with the plate reader. Harvested cells were then washed once in lf-SC (without glucose) and resuspended in lf-SC (without glucose) to an OD_600_ of 0.9. After resuspension, the 0 h sample was analyzed in the plate reader. The cells were then incubated in 15 ml falcons at 30 °C and 150 rpm. 180 µl samples were taken regularly to analyze the pH during starvation in the plate reader (30 °C). After 3.5 h of starvation, 180 µl samples were analyzed for 25 min (30 °C, analysis every 10 min, double orbital shaking of 700 rpm between the measurements). After overall 4 hours of glucose starvation, glucose was added to the microtiter plate to a final concentration of 2% (w/v). The change in fluorescence intensity was analyzed with the Clariostar plate reader (set to 30 °C, analysis every 4 min, double orbital shaking of 700 rpm between the measurements).

For the octanoic acid experiment, biological triplicates were grown overnight in lf-SCD (0 mM methionine) and harvested in the exponential phase (OD_600_ between 1.0 and 2.0). Cells were washed once with and then resuspended in lf-SMD (40 mM KH_2_PO_4_, pH 6.3) to an OD_600_ of 0.9. We chose lf-SMD medium with 40 mM KH_2_PO_4_ because it was able to fully buffer the pH upon addition of 150 mg L^−1^ octanoic acid. After resuspension, 180 µl samples of the cultures were added to a microtiter plate. Octanoic acid was added from a 10x stock to final concentrations of 0, 50, 100, and 150 mg L^−1^. The 10x stocks were prepared from a 40 mM octanoic acid stock solution in 70% ethanol. The ethanol concentration of all 10x stock solutions was kept equal so that the final concentration of ethanol in the medium of all samples was 0.18% (v/v). Immediately after octanoic acid addition the cells were shaken 30 s at 700 rpm and then analyzed in the plate reader.

For all experiments in the Clariostar plate reader, background fluorescence of empty vector controls (CEN.PK2-1C cells transformed with p426MET25, biological triplicates) were also followed over time and substracted as a blank from all raw data.

### Fluorescence Microscopy

CEN.PK2-1C harboring the p426MET-sfpHluorin or the p426MET-ER-sfpHluorin plasmid were grown in lf-SCD to the exponential phase (to an OD_600_ of 1.0 to 2.0). Cells were diluted 1:1 with fresh lf-SCD containing 0.6% low melting agarose (Roth) before applying them to the glass microscope slide. Cells were analyzed with the confocal laser scanning microscope Leica TCS SP5 (Leica Microsystems) with a HCX PL APO lambda blue 63.0 × 1.40 OIL UV objective and excitation at 488 nm and an emission bandwidth of 492 nm to 564 nm.

### Data availability

All data generated or analyzed during this study are included in this published article, its Supplementary Information file or will be made available upon reasonable request.

## Electronic supplementary material

Supplementary Information
