# Supplementary material for: A superfolder variant of pH-sensitive pHluorin for in vivo pH measurements in the endoplasmic reticulum
Source: Sci Rep. 2018 Aug 10;8:11985. doi: 10.1038/s41598-018-30367-z (PMC6086885; doi:10.1038/s41598-018-30367-z)
Supplement: Supplementary file 1 — Supplementary Information [file 41598_2018_30367_MOESM1_ESM.pdf]

Supplementary Information

A superfolder variant of pH-sensitive pHluorin for *in vivo* pH measurements in the endoplasmic reticulum

Mara Reifenrath[1] and Eckhard Boles[1]

[1] Institute of Molecular Biosciences, Faculty of Biological Sciences, Goethe University Frankfurt, Max-von-Laue Straße 9, 60438 Frankfurt am Main, Germany

Figure S1. pH dependence of excitation spectra of cytosolically located, conventional pHluorin The cells were permeabilized with digitonin and resuspended in citric acid/Na2HPO4 buffer of pH values ranging from 5.0 to 9.0 to an OD600 of 0.5. The emission intensity was recorded at 508 nm.

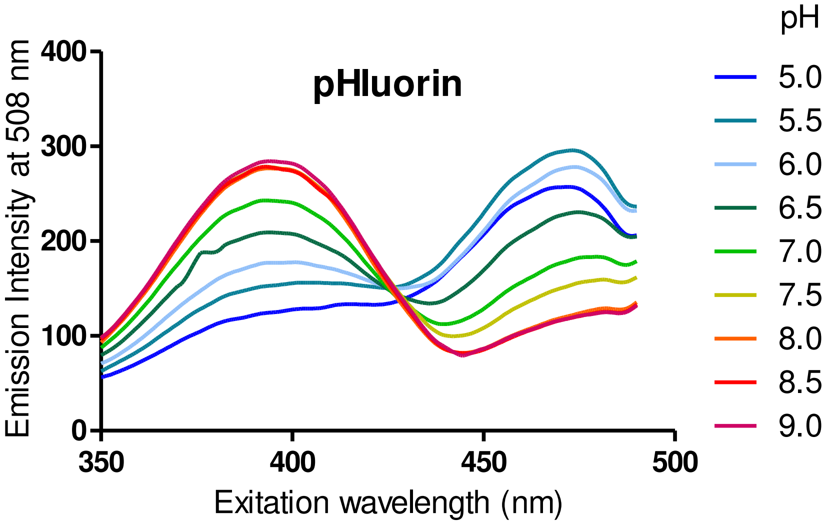

Figure S2. pH calibration and sigmoidal dose response fits for CEN.PK2-1C expressing sfpHluorin (A) and ER-sfpHluorin (B) grown in lf-SCD with 0.1 mM methionine. The graphs show the mean and standard error (mean±s.d.) of biological triplicates.

S5 A)

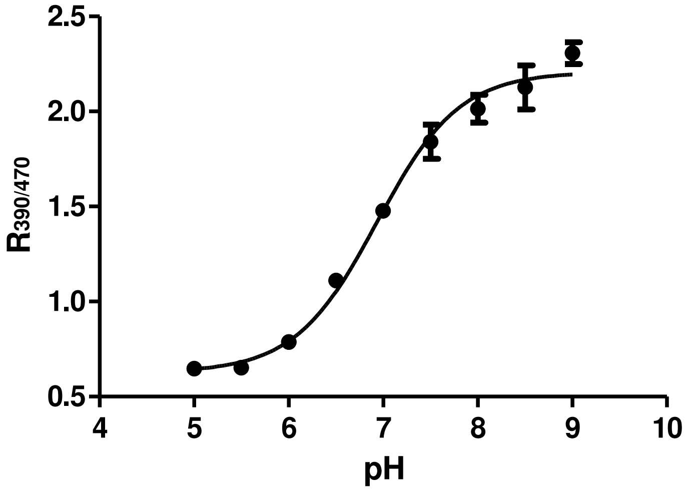

**Best-fit values**	
Bottom	0,6269
Top	2,208
LogEC50	6,934
EC50	8,596e+006
**Std. Error**	
Bottom	0,03009
Top	0,02853
LogEC50	0,04864
**95% Confidence Intervals**	
Bottom	0.5648 to 0.6890
Top	2.150 to 2.267
LogEC50	6.834 to 7.035
EC50	6.822e+006 to 1.083e+007
**Goodness of Fit**	
Degrees of Freedom	24
R^2	0,9866
Absolute Sum of Squares	0,1415
Sy.x	0,07677

S5 B)

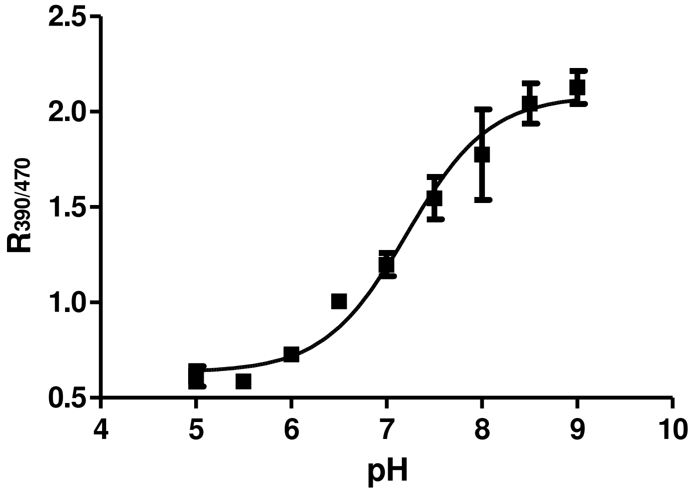

**Best-fit values**	
Bottom	0,6339
Top	2,084
LogEC50	7,212
EC50	1,628e+007
**Std. Error**	
Bottom	0,04070
Top	0,04838
LogEC50	0,08036
**95% Confidence Intervals**	
Bottom	0.5499 to 0.7179
Top	1.984 to 2.184
LogEC50	7.046 to 7.378
EC50	1.111e+007 to 2.385e+007
**Goodness of Fit**	
Degrees of Freedom	24
R^2	0,9643
Absolute Sum of Squares	0,3205
Sy.x	0,1156

Table S1. DNA Sequence of all GFP variants used in this study

Name	DNA sequence	Reference
**Superfolder GPF**	ATGAGCAAAGGAGAAGAACTTTTCACTGGAGTTGTCCCAATTCTTGTTGAATTAGATGG<br>TGATGTTAATGGGCACAAATTTTCTGTCCGTGGAGAGGGTGAAGGTGATGCTACAAACG<br>GAAAACTCACCCTTAAATTTATTTGCACTACTGGAAAACTACCTGTTCCGTGGCCAACAC<br>TTGTCACTACTCTGACCTATGGTGTTCAATGCTTTTCCCGTTATCCGGATCACATGAAAC<br>GGCATGACTTTTTCAAGAGTGCCATGCCCGAAGGTTATGTACAGGAACGCACTATATCT<br>TTCAAAGATGACGGGACCTACAAGACGCGTGCTGAAGTCAAGTTTGAAGGTGATACCCT<br>TGTTAATCGTATCGAGTTAAAGGGTATTGATTTTAAAGAAGATGGAAACATTCTTGGACA<br>CAAACTCGAGTACAACTTTAACTCACACAATGTATACATCACGGCAGACAAACAAAAGA<br>ATGGAATCAAAGCTAACTTCAAAATTCGCCACAACGTTGAAGATGGTTCCGTTCAACTA<br>GCAGACCATTATCAACAAAATACTCCAATTGGCGATGGCCCTGTCCTTTTACCAGACAAC<br>CATTACCTGTCGACACAATCTGTCCTTTCGAAAGATCCCAACGAAAAGCGTGACCACAT<br>GGTCCTTCTTGAGTTTGTAACTGCTGCTGGGATTACACATGGCATGGATGAGCTCTACA<br>AATAA	Pédelacq et al., 2006
**pHluorin**	ATGAGTAAAGGAGAAGAACTTTTCACTGGAGTTGTCCCAATTCTTGTTGAATTAGATGGT<br>GATGTTAATGGGCACAAATTTTCTGTCAGTGGAGAGGGTGAAGGTGATGCAACATACG<br>GAAAACTTACCCTTAAATTTATTTGCACTACTGGAAAACTACCTGTTCCATGGCCAACAC<br>TTGTCACTACTTTCTCTTATGGTGTTCAATGCTTTTCAAGATACCCAGATCATATGAAACG<br>GCATGACTTTTTCAAGAGTGCCATGCCCGAAGGTTATGTACAGGAAAGAACTATATTTTT<br>CAAAGATGACGGGAACTACAAGACACGTGCTGAAGTCAAGTTTGAAGGTGATACCCTTG<br>TTAATAGAATCGAGTTAAAAGGTATTGATTTTAAAGATGATGGAAACATTCTTGGACACA<br>AATTGGAATACAACTATAACGAGCACTTGGTGTACATCATGGCAGACAAACAAAAGAAT<br>GGTACCAAAGCTATCTTTCAAGTTCACCACAACATTGAAGATGGAGGCGTTCAACTAGC<br>AGACCATTATCAACAAAATACTCCAATTGGCGATGGCCCTGTCCTTTTACCAGACAACCA<br>TTACCTGCACACACAATCTGCCCTTTCGAAAGATCCCAACGAAAAGAGAGACCACATGG<br>TCCTTCTTGAGTTTGTAACAGCTGCTGGGATTACACATGGCATGGATGAACTATACAAAT<br>AA	Miesen-böck et al., 1998; Orij et al., 2009
**ER-pHluorin** (SSBiP-sfpHluorin-HDEL)	ATGTTTTTCAACAGACTAAGCGCTGGCAAGCTGCTGGTACCACTCTCCGTGGTCCTGTA<br>CGCCCTTTTCGTGGTAATATTACCTTTACAGAATTCTTTCCACTCCTCCAATGTTTTAGTT<br>AGAGGTAGTAAAGGAGAAGAACTTTTCACTGGAGTTGTCCCAATTCTTGTTGAATTAGA<br>TGGTGATGTTAATGGGCACAAATTTTCTGTCAGTGGAGAGGGTGAAGGTGATGCAACAT<br>ACGGAAAACTTACCCTTAAATTTATTTGCACTACTGGAAAACTACCTGTTCCATGGCCAA<br>CACTTGTCACTACTTTCTCTTATGGTGTTCAATGCTTTTCAAGATACCCAGATCATATGAA<br>ACGGCATGACTTTTTCAAGAGTGCCATGCCCGAAGGTTATGTACAGGAAAGAACTATAT<br>TTTTCAAAGATGACGGGAACTACAAGACACGTGCTGAAGTCAAGTTTGAAGGTGATACC<br>CTTGTTAATAGAATCGAGTTAAAAGGTATTGATTTTAAAGATGATGGAAACATTCTTGGA<br>CACAAATTGGAATACAACTATAACGAGCACTTGGTGTACATCATGGCAGACAAACAAAA<br>GAATGGTACCAAAGCTATCTTTCAAGTTCACCACAACATTGAAGATGGAGGCGTTCAAC<br>TAGCAGACCATTATCAACAAAATACTCCAATTGGCGATGGCCCTGTCCTTTTACCAGACA<br>ACCATTACCTGCACACACAATCTGCCCTTTCGAAAGATCCCAACGAAAAGAGAGACCAC<br>ATGGTCCTTCTTGAGTTTGTAACAGCTGCTGGGATTACACATGGCATGGATGAACTATA<br>CAAATTCGAACACGACGAATTGTAG	This study

**Superfolder pHluorin**<br>(sfpHluorin)	ATGAGCAAAGGAGAAGAACTTTTCACTGGAGTTGTCCCAATTCTTGTTGAATTAGATGG<br>TGATGTTAATGGGCACAAATTTTCTGTCCGTGGAGAGGGTGAAGGTGATGCTACAAACG<br>GAAAACTCACCCTTAAATTTATTTGCACTACTGGAAAACTACCTGTTCCGTGGCCAACAC<br>TTGTCACTACTCTGTCTTATGGTGTTCAATGCTTTTCCCGTTATCCGGATCACATGAAAC<br>GGCATGACTTTTTCAAGAGTGCCATGCCCGAAGGTTATGTACAGGAACGCACTATATCT<br>TTCAAAGATGACGGGACCTACAAGACGCGTGCTGAAGTCAAGTTTGAAGGTGATACCCT<br>TGTTAATCGTATCGAGTTAAAGGGTATTGATTTTAAAGAAGATGGAAACATTCTTGGACA<br>CAAACTCGAGTACAACTTTAACGAGCACTTGGTATACATCACGGCAGACAAACAAAAGA<br>ATGGAACCAAAGCTATCTTCCAAGTTCACCACAACGTTGAAGATGGTTCCGTTCAACTA<br>GCAGACCATTATCAACAAAATACTCCAATTGGCGATGGCCCTGTCCTTTTACCAGACAAC<br>CATTACCTGCACACACAATCTGTCCTTTCGAAAGATCCCAACGAAAAGCGTGACCACAT<br>GGTCCTTCTTGAGTTTGTAACTGCTGCTGGGATTACACATGGCATGGATGAGCTCTACA<br>AATAA	This study
**ER-Superfolder pHluorin**<br><br>(SSBiP-sfpHluorin-HDEL)	ATGTTTTTCAACAGACTAAGCGCTGGCAAGCTGCTGGTACCACTCTCCGTGGTCCTGTA<br>CGCCCTTTTCGTGGTAATATTACCTTTACAGAATTCTTTCCACTCCTCCAATGTTTTAGTT<br>AGAGGTAGCAAAGGAGAAGAACTTTTCACTGGAGTTGTCCCAATTCTTGTTGAATTAGA<br>TGGTGATGTTAATGGGCACAAATTTTCTGTCCGTGGAGAGGGTGAAGGTGATGCTACAA<br>ACGGAAAACTCACCCTTAAATTTATTTGCACTACTGGAAAACTACCTGTTCCGTGGCCAA<br>CACTTGTCACTACTCTGTCTTATGGTGTTCAATGCTTTTCCCGTTATCCGGATCACATGA<br>AACGGCATGACTTTTTCAAGAGTGCCATGCCCGAAGGTTATGTACAGGAACGCACTATA<br>TCTTTCAAAGATGACGGGACCTACAAGACGCGTGCTGAAGTCAAGTTTGAAGGTGATAC<br>CCTTGTTAATCGTATCGAGTTAAAGGGTATTGATTTTAAAGAAGATGGAAACATTCTTGG<br>ACACAAACTCGAGTACAACTTTAACGAGCACTTGGTATACATCACGGCAGACAAACAAA<br>AGAATGGAACCAAAGCTATCTTCCAAGTTCACCACAACGTTGAAGATGGTTCCGTTCAA<br>CTAGCAGACCATTATCAACAAAATACTCCAATTGGCGATGGCCCTGTCCTTTTACCAGAC<br>AACCATTACCTGCACACACAATCTGTCCTTTCGAAAGATCCCAACGAAAAGCGTGACCA<br>CATGGTCCTTCTTGAGTTTGTAACTGCTGCTGGGATTACACATGGCATGGATGAGCTCT<br>ACAAATTCGAACACGACGAATTGTAG	This study

Table S2. Plasmids used in this study

Plasmids	Characteristics	Reference
**Plasmids used for fermentations**		
p426MET25	2µ, *URA3, Amp*^{r}, *MET25*$_{pr}$, *CYC1*$_{ter}$	(Mumberg et al., 1995)
MRV55	p426MET25, sfpHluorin	This study
MRV65	p426MET25 SSKar2-sfpHluorin-HDEL (ER-pHluorin)	This study
MRV66	p426MET25 SSKar2-sfpHluorin-HDEL (ER-sfpHluorin)	This study

Table S3. Primers used in this study

Primer name	Sequence 5‘-3‘	Application
MOP291	GCGTCTGTTAGAAAGGAAGTTTTTCC	Binding in *MET25*$_{prom}$, sequencing, forward
MOP290	ACCTAGACTTCAGGTTGTC	Binding in *CYC1*$_{term}$, sequencing, reverse
MRP57	AATTCTATTACCCCCATCCATACTCTAGAAATGAGTAAAGGAGAAGAACTTTTC	Binding *pHluorin*, overhang to *MET25*$_{prom}$, forward
MOP252	TAAGCGTGACATAACTAATTACATGACTCGAGTTATTTGTATAGTTCATCCATGC	Binding *pHluorin*, overhang to *CYC1*$_{term}$, reverse
JTP96	AATTCTATTACCCCCATCCATACTCTAGAAATGAGCAAAGGAGAAGAACTTTTC	Binding *superfolderGFP*, overhang to *MET25*$_{prom}$, forward
MRP85	TTGAACACCATAAGACAGAGTAGTGACAAGTGTTGG	Introducing mutation T65S, reverse
MRP86	CTTGTCACTACTCTGTCTTATGGTGTTCAATGCTTTTCCC	Introducing mutation T65S, reverse
MRP87	TCCATTCTTTTGTTTGTCTGCCGTGATGTATACCAAGTGCTCGTTAAAGTTGTACTCGAGTTTGTG	Introducing mutation S147E, N149L, reverse
MRP88	TACATCACGGCAGACAAACAAAAGAATGGAACCAAAGCTATCTTCCAAGTTCACCACAACGTTGAAGATGGTTC	Introducing mutation I161T, N164I, K166Q, I167V, R168H, forward
MRP89	AGGACAGATTGTGTGTGCAGGTAATGGTTGTCTGGTAAAAG	Introducing mutation S202H, reverse
MRP90	CAACCATTACCTGCACACACAATCTGTCCTTTCGAAAG	Introducing mutation S202H, forward
JRP97	AATGTAAGCGTGACATAACTAATTACATGATTATTTGTAGAGCTCATCCATGC	Binding *superfolderGFP*, overhang to *CYC1*$_{term}$, reverse
MRP07	AATTCTATTACCCCCATCCATACTCTAGAAATGTTTTTCAACAGACTAAGC	Binding KAR2 signal sequence, overhang to *MET25*$_{prom}$, forward
JTP89	ACCTCTAACTAAAACATTGGAGGAGTGGAAAG	Binding KAR2 signal sequence, reverse
JTP92	CTTTCCACTCCTCCAATGTTTTAGTTAGAGGTAGCAAAGGAGAAGAACTTTTC	Binding superfolder GFP/pHluorin, overhang to KAR2 signal sequence, forward
JRP93	GACTACAATTCGTCGTGTTCGAATTTGTAGAGCTCATCCATGC	Binding superfolder GFP/pHluorin, introducing ER-retention tag, reverse
MRP06	AATGTAAGCGTGACATAACTAATTACATGACTACAATTCGTCGTGTTCG	Binding ER-retention tag, overhang to *CYC1*$_{term}$, reverse

Table S4. Sigmoidal dose-response fits

A) sfpHluorin (0 mM methionine)

**Best-fit values**	
Bottom	0,6462
Top	2,316
LogEC50	6,929
EC50	8,488e+006
**Std. Error**	
Bottom	0,03232
Top	0,03051
LogEC50	0,04939
**95% Confidence Intervals**	
Bottom	0.5795 to 0.7129
Top	2.253 to 2.379
LogEC50	6.827 to 7.031
EC50	6.712e+006 to 1.073e+007
**Goodness of Fit**	
Degrees of Freedom	24
R^2	0,9862
Absolute Sum of Squares	0,1624
Sy.x	0,08227

B) ER-sfpHluorin (0 mM methionine)

**Best-fit values**	
Bottom	0,5025
Top	2,240
LogEC50	7,273
EC50	1,874e+007
**Std. Error**	
Bottom	0,05035
Top	0,06296
LogEC50	0,08509
**95% Confidence Intervals**	
Bottom	0.3986 to 0.6064
Top	2.110 to 2.370
LogEC50	7.097 to 7.448
EC50	1.251e+007 to 2.809e+007
**Goodness of Fit**	
Degrees of Freedom	24
R^2	0,9601
Absolute Sum of Squares	0,5114
Sy.x	0,1460
